# Supplementary material for: Real-time racial discrimination, affective states, salivary cortisol and alpha-amylase in Black adults
Source: PLoS One. 2022 Sep 14;17(9):e0273081. doi: 10.1371/journal.pone.0273081 (PMC9473392; doi:10.1371/journal.pone.0273081)
Supplement: Attachment — Submitted filename: Review.docx [file pone.0273081.s004.docx]

I’ve completed my review PONE-D-22-05171 “Real-time racial discrimination, affective states, salivary cortisol and alpha-amylase in Black adults”. The authors, using longitudinal data from 12 health Black adults, examined the association between racial discrimination and stress response system (cortisol and alpha amylase). The findings revealed that previous day racial discrimination was associated with cortisol; also, microaggressions was associated with cortisol during the same day. Although this study moves research on racial discrimination and objective measures of stress response forward, there is an issue of concern worth noting.

I would like for the authors to connect racism, specifically antiBlack racism, to discrimination. In the current form, discrimination can be reduced to a few “bad apples” instead of a larger system of racism, especially how discrimination and microaggression. For example, if racism conceptualized as both ideology (e.g., attitudes about race and racial inequality) and structure (e.g., laws, policies, etc.), then discrimination can be framed as part of the larger system of racism. This context should be made apparent in both the introduction and the discussion. To help the authors accomplish this goal, I recommend the following papers, especially the Golash-Boza paper:

Doane, A. (2017). Beyond color-blindness:(Re) theorizing racial ideology. *Sociological Perspectives*, *60*(5), 975-991.

Golash-Boza, T. (2016). A critical and comprehensive sociological theory of race and racism. *Sociology of race and ethnicity*, *2*(2), 129-141.
