# Supplementary material for: Real-time racial discrimination, affective states, salivary cortisol and alpha-amylase in Black adults
Source: PLoS One. 2022 Sep 14;17(9):e0273081. doi: 10.1371/journal.pone.0273081 (PMC9473392; doi:10.1371/journal.pone.0273081)
Supplement: Attachment — Submitted filename: Responses to Reviewers.docx [file pone.0273081.s005.docx]

**Responses to the reviewer** We thank the reviewers for the thoughtful reviews. The comments from the reviewers were helpful to strengthen our manuscript.

| **Reviewer 1’s comments** | **Response to the reviewer** |
| --- | --- |
| **Abstract**  1. Beta weights and p values are given, however, these are not necessary or particularly descriptive. All p values are assumed to be significant. An r or R^2 would be easier to interpret. | We calculated partial correlations based on the coefficients in multilevel models and added them to the abstract. |
| **Introduction**  2. It is said that racial discrimination is found to dysregulate the ANS or HPA axis. However, is there evidence for “dysregulation” per se? Dysregulation typically implies a long-term disruption in the capacity for the HPA axis to sufficiently or appropriately activate and restore homeostasis (such as the loss of HPA activity in general adaptation syndrome wherein chronic activation blunts responsivity). It should be clarified that acute insults “activate” the HPA axis and evidence should be provided as to whether a lifetime of racial bias truly “dysregulates” the HPA axis. | We agree with the reviewer. With the current study design, we cannot claim the dysregulation with a relatively short time observation. Throughout the paper, we have removed the word “dysregulation” when we meant a HPA activity or acute reactivity. |
| 3. The reasons for sAA to reflect ANS function should be described (i.e. parotid stimulation by locus coeruleus). | We have added the reasons for sAA to reflect the ANS function as suggested. |
| 4. It is argued by authors that one of the reasons prior literature has not found a relationship between ANS or HPA axis biomarkers is that studies have been retrospective, thereby accounting for a lifetime of racial bias as opposed to real-time assessment. If it is the case that lifetime racial experiences do not predict ANS or HPA axis function, then this would seem to argue against a true “dysregulation” of the ANS and HPA axis in favor of more immediate changes that may be reversible. Authors should consider this in their interpretation. | We have clarified the texts by specifying the role of the measurement timeframe in prior research in establishing the temporal relationship and by eliminating the word “dysregulation” to be appropriate in the context. |
| 5. Why was a blunted wakening cortisol response hypothesized when it has been previously observed that peer rejection increases wakening cortisol? | We agree with the reviewer. We have revised the hypotheses by specifying directional and exploratory, nondirectional hypotheses, given the inconsistent prior research findings. |
| **RESULTS/DISCUSSION** 6. Fig. 1 needs to include a measure of variance for each timepoint. | We have added standard errors for each timepoint. |
| 7. The correlation with sex is a confusing to interpret. Typically, discrete variables such as sex are not appropriate for continuous inferentials. Sex should really be assessed via t-test. It is suggested to remove the current correlations for this variable in favor of t-tests. | We agree with the reviewer. We have conducted Wilcoxon’s test due to the small sample size and revised the table accordingly. |
| 8. “Dysregulation” of the HPA axis is said to be associated with poor health outcomes, but again this wording should be justified. | The HPA axis is dysregulated in a wide range of physiological and pathological conditions, including chronic exposure to stress. Thus, we have eliminated the word “dysregulation” to avoid confusion, specified parameters (e.g., flatter diurnal slope) and provided contexts. |
| 9. It would be useful to include the “stress from daily hassles” in a supplement. | Source of stress from daily hassles varied by participants and days. We did not find any meaningful or significant relationships with dependent variables or other covariates. |
| 10. Authors should be careful not to over-interpret cortisol measures. For example, higher salary was associated with. | Since the finding on salary was based on bivariate analysis, and it was no longer significant in the multilevel model, we have deleted it from the main texts (remains in Table 2). We also emphasized that some of the significant variables in the bivariate analysis became insignificant in multilevel models. |
| 11. Did authors consider that recording microaggressions might heighten awareness to them? Some reactivity to the recording process would be expected, perhaps increasing the HPA axis effect of microaggressions in the current study. This should be considered/discussed. | In the limitation section, we have added the potential influences of the repetitive assessment on the HPA and ANS activities. |
| Minor comments: 1. Some grammatical errors are noted throughout the manuscript (e.g., inappropriate tense, missing articles such as “the” or “a”, etc). The manuscript should be carefully proofread prior to resubmission. 2. In Table 1, it should read equal to or greater than the top values for salary and BMI. | We have corrected grammatical errors and the *equal to greater* signs in income and BMI in Table 1. |
| **Reviewer 2’s comments** | **Response to the reviewer** |
| I would like for the authors to connect racism, specifically antiBlack racism, to discrimination. In the current form, discrimination can be reduced to a few “bad apples” instead of a larger system of racism, especially how discrimination and microaggression. For example, if racism conceptualized as both ideology (e.g., attitudes about race and racial inequality) and structure (e.g., laws, policies, etc.), then discrimination can be framed as part of the larger system of racism. This context should be made apparent in both the introduction and the discussion. | Thank you for providing the helpful articles. We agree with the reviewer that interpersonal level-racial discrimination is deeply rooted in system-level social inequalities and racial discrimination. In the introduction and discussion sections, we have added the context of racial discrimination as part of the societal level-racism. |

NOTE: Newly added citations are:# 1-5, 11-15
